# TECHNOLOGY IN OLDER ADULTS

**DOI:** 10.1093/geroni/igae098.1076

**Published:** 2024-12-31

## Abstract

Use of technology to improve health of older adults

